# Ranking the invasions of cheaters in structured populations

**DOI:** 10.1038/s41598-020-59020-4

**Published:** 2020-02-10

**Authors:** Guoli Yang, Matteo Cavaliere, Cheng Zhu, Matjaž Perc

**Affiliations:** 1 Unit 66136, Beijing, 100042 China; 2 Science and Technology on Information Systems Engineering Laboratory, National University of Defense Technology, Changsha, 410073 China; 3 Department of Computing and Mathematics, Manchester Metropolitan University, Manchester, United Kingdom; 4 Faculty of Natural Sciences and Mathematics, University of Maribor, Koroška cesta 160, 2000 Maribor, Slovenia; 5 Department of Medical Research, China Medical University Hospital, China Medical University, 404 Taichung, Taiwan; 6 Complexity Science Hub Vienna, Josefstädterstraße 39, 1080 Vienna, Austria

**Keywords:** Applied physics, Evolutionary theory

## Abstract

The identification of the most influential individuals in structured populations is an important research question, with many applications across the social and natural sciences. Here, we study this problem in evolutionary populations on static networks, where invading cheaters can lead to the collapse of cooperation. We propose six strategies to rank the invading cheaters and identify those which mostly facilitate the collapse of cooperation. We demonstrate that the type of successful rankings depend on the selection strength, the underlying game, and the network structure. We show that random ranking has generally little ability to successfully identify invading cheaters, especially for the stag-hunt game in scale-free networks and when the selection strength is strong. The ranking based on degree can successfully identify the most influential invaders when the selection strength is weak, while more structured rankings perform better at strong selection. Scale-free networks and strong selection are generally detrimental to the performance of the random ranking, but they are beneficial for the performance of structured rankings. Our research reveals how to identify the most influential invaders using statistical measures in structured communities, and it demonstrates how their success depends on population structure, selection strength, and on the underlying game dynamics.

## Introduction

In evolutionary structured populations, individuals usually adopt the behaviors held by their neighbours, leading to the propagation of states throughout the network. Models of evolutionary games on networks have been studied for a long time^1–6^, with the dynamics of competing strategies studied using evolutionary game theory where the fitness of an individual is dependent on the neighbourhood configuration and the game settings. A particular case of interest is the conflict between cooperative and cheating individuals^6^. In this case, few initial (cheating) players (just like the initial spreaders in propagation networks) in a cooperative community can spread and lead to a full cheater invasion and the consequent collapse of cooperation. In this paper we address the question on how to identify the most influential cheaters, i.e., those which most probably will lead to the collapse of cooperation, in structured populations.

In order to find a set of initial spreaders which can achieve the most prevalent propagation collectively, previous works have tried to define some statistical measures to identify the influential spreaders. It is known that the influence of a node is highly dependent on the network structure and its location on the network, but how to calculate the actual relevance of a node is not trivial. Despite there are more than 30 methods to characterize the *importance* of a node, there is not an ubiquitous one which can be applied to different kinds of dynamical systems. Generally speaking, there are four types of ranking measurements focused on the identification of vital nodes^7^ which can be divided into rankings based on local information, rankings based on global information, rankings based on iterative refinement, and rankings based on node removal. In the family of rankings based on local information, degree centrality^8^ is the most popular one, but is not able to reflect details for the higher order neighbours. Possible extensions are the neighbourhood connectivity and clustering coefficient through LocalRank^9^ and ClusterRank^10^. To obtain the position of the nodes, one can decompose the network gradually to compute the coreness of the nodes^11^. As this k-shell decomposition is highly coarse-grained, i.e., nodes in same shell may be very different, mixed degree decomposition^12^ and VoteRank^13^ have been proposed to distinguish the links connecting to removed nodes and to remaining nodes. In the case of rankings based on global information, the path from one node to another is also taken into account. Eccentricity^14^, closeness centrality^15^, Katz centrality^16^ etc. are calculated to characterize the diameter of the network, the average shortest path distance and the overall influence of all paths. In particular, betweenness centrality^17^ has been extensively studied^18–20^ as it measures the role of *bridges* in the network controlling the flow of information. In the case of rankings based on iterative refinement, the *importance* of a node is determined by the mutual enhancement effect under the framework of Markovian dynamics, where every node gets support from its in-neighbours and then gives support to the out-neighbours. Algorithms include eigenvector centrality^21^, Alpha centrality^22^, cumulative nomination^23^, PageRank^24^, HITs^25^ etc. As one of the most well-known methods to rank websites, PageRank evaluates the *importance* of a node by the quantity and quality of the pages linked to it. Considering that the dynamics of visiting nodes is triggered by random walk and a dangling node will always gain values and never give them out, random jumping-out and LeaderRank^26,27^ were explored to avoid such drawback. The last type of ranking methods is based on node removal^28,29^, whose main idea is that more influential nodes will highly affect the connectivity or distance of the network when they are removed. In this case, the typical measurements to evaluate the changes of function and structure of the networks are the shortest path distance^30^, the number of spanning trees^31^, and the contraction of network^32^. All these types of ranking methodologies are generally applied to spreading models that are based on the stochastic triggering of interactions from one state to another between connecting individuals, including independent cascade model, linear threshold model, epidemic model (such as SI, SIS, SIR), and voter models^33^. However, when the spreading dynamics is coupled to evolutionary dynamics under the framework of evolutionary game theory (EGT), things may dramatically change as the underlying competition can lead to complicated dynamics in the identification of influential nodes which obtain a fitness (driving their reproduction or disappearance) based on their own and their neighbours strategies. This paper, for the first time to our knowledge, explores the possibility of using some of the previously investigated rankings to identify the most successful invaders in evolutionary graph theory^34^, exploring how the success of different rankings depend on the game dynamics, selection strength and network structure.

## Evolutionary Graph Theory

In evolutionary graph theory^34^, the players (individuals) occupy the vertices of a graph (network), and the connections between each other indicate the possible interactions. The graph (population structure) is fixed during the evolutionary dynamics, and the size of the population is constant. In our scenario, the players can use one of the two strategies: cooperation (*C*) and defection (*D*), that is also called cheating. In a population of *N* individuals consisting of cooperators and defectors, the total payoff of a player is the sum of the payoffs obtained by interacting with each one of the neighbours. The payoff matrix which represents the payoff for each possible type of interaction is:1We use this payoff matrix to study the conflict between cooperators (C) and defectors (cheaters, D) in four different types of games (by fixing $R=1$, $P=0$, and opportunely varying *S* and *T* we can obtain stag-hunt, snow-drift, harmony and prisoner-s dilemma game^35^).

The *fitness* of a player is given by a constant term - baseline fitness - plus the payoff obtained from the interactions. Therefore, the fitness of a player is defined as:

2 $${f}_{i}=1-w+w{\pi }_{i}$$

where *w* is the intensity of selection.

**Figure 1:**
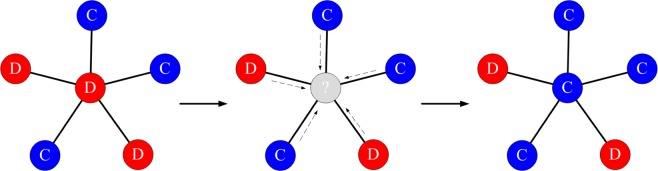
The evolutionary dynamics of a death-birth model. At each update step, each individual occupies the vertex of a graph and derives a payoff from the interactions with its neighbours (the payoff obtained for each interaction is given by the payoff matrix of the game). A random node is selected to die and the neighbours compete to occupy the empty site with probability proportional to their fitness.

Strategies that do well are more likely to be imitated by others. In this paper we use the standard ‘death-birth’ model^34^. At each update step, a random node *i* is selected for death and the neighbours compete to occupy the empty site with probability proportional to their fitness. The evolutionary dynamics can be defined by a discrete sequence of update steps as shown in Fig. 1.

In this paper we use the above described model of evolutionary graph theory to explore the identification of the most influential cheating (defecting) nodes, i.e., those with higher chances of leading to a full cheaters invasion and cooperation collapse.

Among the ones discussed in the Introduction, in this paper we focus on the following ranking strategies which establish different ways to select the *top-n* cheating nodes in a population of cooperators (i.e., the initial invaders). In general, two types of nodes are considered in the network, one is the type of **seed nodes**, selected into the *top-n-list* as initial defectors; and the other type is that of **potential nodes**, which is the types of the nodes not selected into the *top-n-list*.

The idea is to pick *n* nodes as defectors (the so-called *top-n* nodes), in an initial network of all cooperators, and see whether they will survive and spread in the population leading to a complete defectors invasion. The initial density of defectors is defined by $\rho =n$/*N*. Depending on the type of ranking, the initial *n* defectors are selected in different ways.

We investigate the following rankings which describe different ways to select the *top-n* nodes (see also the Methods section)

- random ranking: the *top-n* nodes are randomly chosen nodes in the network and selected to be defectors.
- degree ranking: the *top-n* nodes are selected to be defectors according to their degree (i.e., higher is the node degree, higher the chances that the node is selected).
- betweenness ranking: the *top-n* nodes are selected to be defectors according to their betweenness (i.e., higher is the betweenness of a node, higher is the chance that the node is selected).
- k-shell ranking: the *top-n* nodes are selected to be defectors based on their k-shell^11^ (higher the k-shell of a node, higher the chances that the node is selected).
- N-weighted degree ranking: the *top-n* nodes are selected to be defectors based on the negative weighted degree^36^.
- P-weighted degree ranking: the *top-n* nodes are selected to be defectors based on the positive weighted degree^36^.

In this paper we refer to all the rankings except the random one as structured rankings, as they imply the addition of invaders using some kind of strategic choice based on the population structure.

## The Initial Position of Defectors Matters

**Figure 2:**
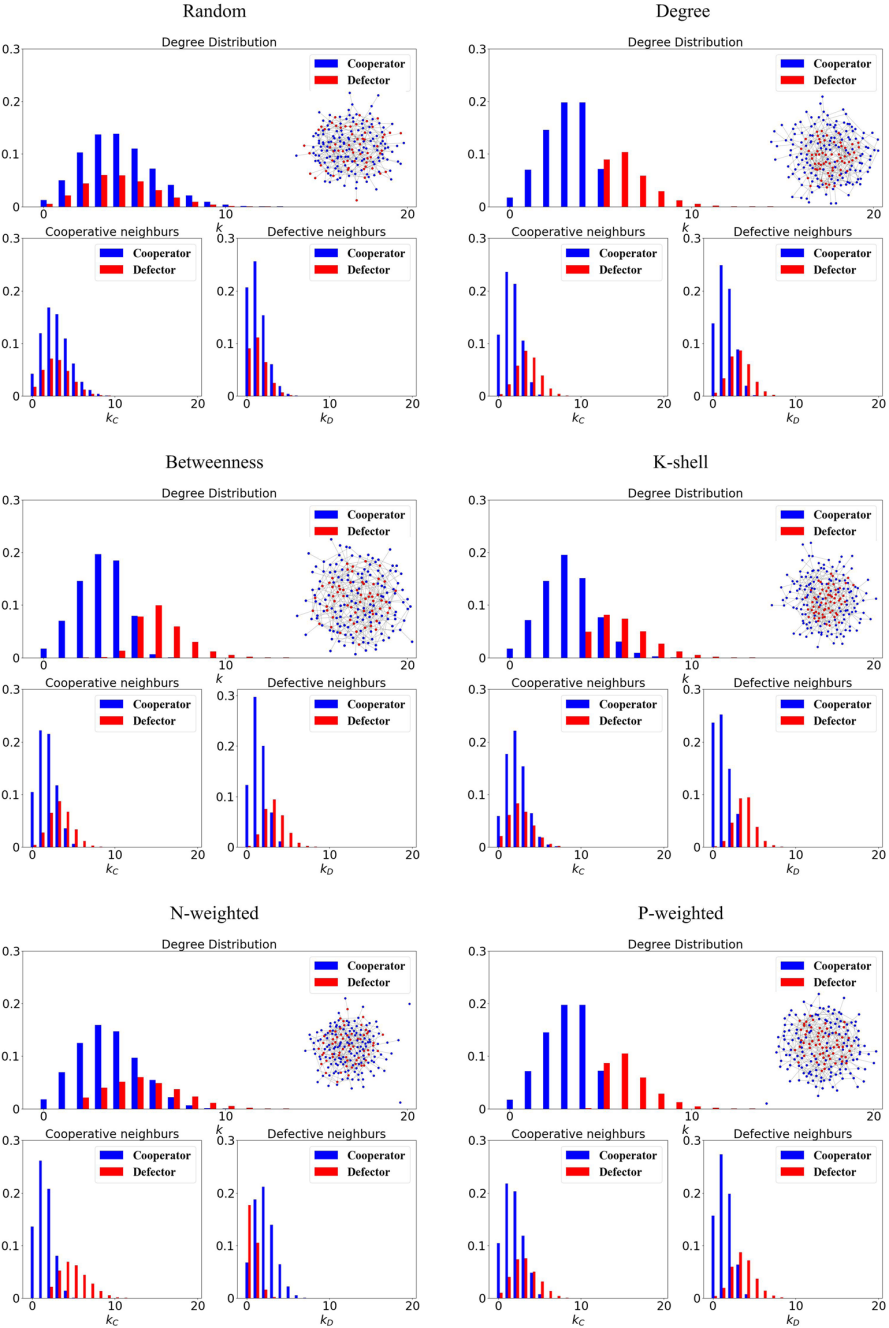
Rankings and initial positions of invading cheaters. We show how in an ER random network, the different rankings affect the initial positions of the cheaters. We plot the initial degree and neighbours distributions for cooperators and defectors. We use an initial density of cheaters $\rho =30 \%$ nodes, which are nodes to be selected as defectors according to the ranking strategies based on random, degree, betweenness, k-shell, N-weighted degree and P-weighted degree.

To study the success of different rankings we simulate the dynamics of a population of cooperators where some nodes (the *top-n* nodes) are selected as defectors (cheaters) according to the chosen ranking strategy described in the previous Section. We use an initial density of cheaters $\rho =30 \%$ (i.e., 30% of the nodes in the population are initially selected as defectors). To provide an intuition on how the ranking affects the initial positions of the cheaters we plot in Fig. 2 the initial network configurations obtained for the different ranking strategies in the case of an ER random network^37^ with $N=200$ nodes and $E=400$ edges. As we can see, the initial positions of the defectors depend crucially on the chosen ranking. Defectors are more likely to get closer when the invaders are established using a ranking based on degree, k-shell, P-weighted degree. However, when the invaders are established using a ranking based on random or N-weighted degree, the initial defectors tend to be more distant and avoid the overlap of their sphere of influence. To quantify this intuition, we show in Fig. 2 the distributions of cooperators and cheaters using the degree (*k*), the number of cooperative neighbours (*k*_*C*_) and the number of cheating neighbours (*k*_*D*_). Interestingly, an evident distinction is present for the different ranking strategies. The degree distributions for cooperators and defectors are overlapping for the rankings based on random and N-weighted degree, while they are separated for the rankings based on betweenness, k-shell, degree, and P-weighted degree. Furthermore, we can see that the defectors have more cooperative neighbours than the cooperators for the rankings based on N-weighted degree; on the contrary, the cooperators hold more defective neighbours than the defectors. However, for the rankings based on the degree, betweenness and P-weighted degree, the defectors have more defective and cooperative neighbours than the cooperators. As we can see, the defectors selected by using a ranking based on P-weighted degree or degree are more likely to get closer establishing many connections to cooperators.

## The Efficacy of Random Ranking

The different positioning of the invading cheaters in the population structures (which we have intuitively discussed in Fig. 2) can affect the possibility of a cheaters invasion and cooperation collapse (in fact, mixed connections are generally detrimental for cooperators and advantageous for cheaters). However the actual chances of a cooperation collapse may depend on a complex interplay of the underlying game dynamics, network structure and selection strength. To investigate the effects of different games, we fix $R=1$ and $P=0$ and vary *S* from $[\,-\,1,1]$ and *T* from $[0,2]$; in this way we can easily explore different types of games^35^, including stag-hunt game ($S\in [\,-\,1,0]$ and $T\in [0,1]$), prisoner’s dilemma ($S\in [\,-\,1,0]$ and $T\in [1,2]$), harmony game ($S\in [0,1]$ and $T\in [0,1]$) and snowdrift game ($S\in [0,1]$ and $T\in [1,2]$).

We study how the probability of collapse of cooperation is affected by the values of *S* and *T*, by the selection strengths *w*, by the network structures and by the different rankings, i.e., the different ways to add the initial cheating invaders. The initial addition of cheaters can only lead to two possible outcomes - a cooperation collapse, i.e., the initial cheaters spread and fully invade the network; or a cooperation recovery, i.e., the initial cheaters do not spread and the network becomes composed of only cooperators. Here, we use the *probability of collapse*, which is computed as the ratio between the number of cooperation collapses obtained after the addition of the initial cheaters and the total number of simulations.

**Figure 3:**
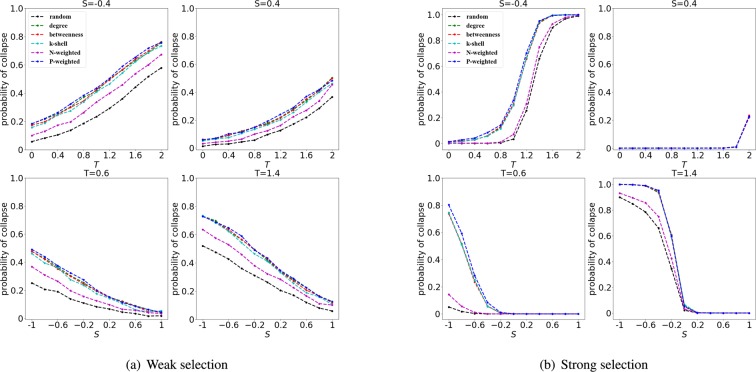
The probability of collapse in random networks at weak and strong selection. The simulations are run with $\rho =30 \%$ nodes selected to be defectors according to the following ranking strategies: random, degree, betweenness, k-shell, N-weighted degree and P-weighted degree. In the upper panels, we use *S* as $S=-\,0.4$ and $S=0.4$, and the x-axis indicates $T\in [0,2]$. In the lower panels the game parameter *T* is fixed as $T=0.6$ and $T=1.4$, and the x-axis indicates the parameter $S\in [\,-\,1,1]$. The strength of selection is $w=0.01$ (left panels) and $w=0.1$ (right panels). Simulations are done using ER random networks and the probability of collapse is obtained using 2000 independent simulations.

In Fig. 3 we present the probability of (cooperation) collapse at weak selection ($w=0.01$) and strong selection ($w=0.1$) for an ER random network with $N=200$ nodes and $E=400$ edges. We consider $S=-\,0.4$ and $S=0.4$, with the values of *T* varying between 0 and 2; and $T=0.6$ and $T=1.4$ with the values of *S* varying between −1 and 1; in this way, we can evaluate the performance for different ranking strategies and for different games.

As we can see in Fig. 3, when the population is structured on an ER network, the different ways to add the initial defectors will lead to quite different outcomes. For fixed *S*, the probability of collapse is enhanced with the increase of *T*, i.e., the temptation to defect helps the propagation of defectors. On the contrary, for fixed *T*, the probability of collapse decreases with the increase of *S* (i.e., a decrease in *S* indicates a higher loss for a cooperator connected to a defector). It is important to notice that the random ranking does not have generally a good performance (we indicate by performance of a ranking its ability to cause a cooperation collapse which also denotes its ability to identify/position successful invaders in the population). In particular, we can see in Fig. 3 that there are several regimes where the rankings based on the degree or the P-weighted degree can outperform the random ranking.

## Most Successful Rankings

While in Fig. 3 we have focused our attention on random networks, the probability of cooperation collapse is highly dependent on the network structure. Depending on the different network structure of the population (we consider lattice, small-world^38^, random and scale-free networks^39^ in the following), the performance of certain rankings can dramatically change.

In order to shed light on the optimal ranking for different scenarios, we compare the above 6 rankings for the harmony game (using $S=0.4$, $T=0.6$), the snowdrift game (using $S=0.4$, $T=1.4$), the stag-hunt game (using $S=-\,0.4$, $T=0.6$) and the prisoner’s dilemma (using $S=-\,0.4$, $T=1.4$).

**Figure 4:**
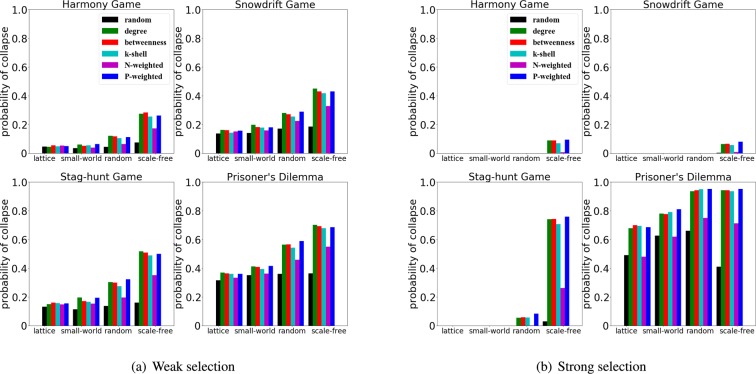
Network structures and selection strength affect the success of the rankings. We use an initial density of cheaters $\rho =30 \%$, and plot the probability of collapse obtained at weak selection ($w=0.01$, left panel) and strong selection ($w=0.1$, right panel). For each subfigure, the x-axis indicates the type of network - lattice, small-world, random and scale-free, while the y-axis indicates the probability of collapse. We test the different rankings (random, degree, betweenness, k-shell, N-weighted and P-weighted) for four typical games.

To study the consequences of network structures and selection strength on the rankings success, we illustrate the probability of collapse for the different rankings in Fig. 4. When the selection strength is weak, the difference between the performance of the different rankings is small, see Fig. 4(a), and the collapse of cooperation is mostly evident for the prisoner’s dilemma in scale-free networks due to the large payoff difference between cooperators and defectors. However, when selection strength is strong, the large payoff obtained by the defectors will be amplified and the difference of performance between the rankings is more evident, especially for the stag-hunt game (Fig. 4(b)).

Figure 4 stresses that the performance of the different rankings depends crucially on the network structures, selection strength and specific games. We can observe that scale-free networks facilitate the collapse of cooperation, in particular when it is used a ranking which tend to select the “hubs” of the network as initial cheaters.

We can also observe that the influence of selection strength is also non-trivial. In fact, a strong selection strength promotes the collapse of cooperation in the prisoner’s dilemma, but is detrimental to the collapse of cooperation in the other regimes. Notably, we can observe that for the stag-hung game (with $S=-\,0.4$ and $T=0.6$), the performance of the rankings for lattice, small-world networks and random networks is generally worse at strong selection than that at weak selection; however, for scale-free networks the scenario is opposite - at strong selection rankings can be more effective than in weak selection due to the advantage provided to defectors placed in “hubs” positions, and selected by the opportune ranking strategies.

If we analyze the performance of each individual ranking strategy, we can observe that the rankings based on P-weighted, degree, betweenness and k-shell generally outperform the ranking based on N-weighted and random, especially in the stag-hunt game. Interestingly, the selection strength can determine the most successful ranking: the ranking based on the degree can outperform the other rankings at weak selection, but the ranking of P-weighted degree works better at strong selection (Fig. 4).

**Figure 5:**
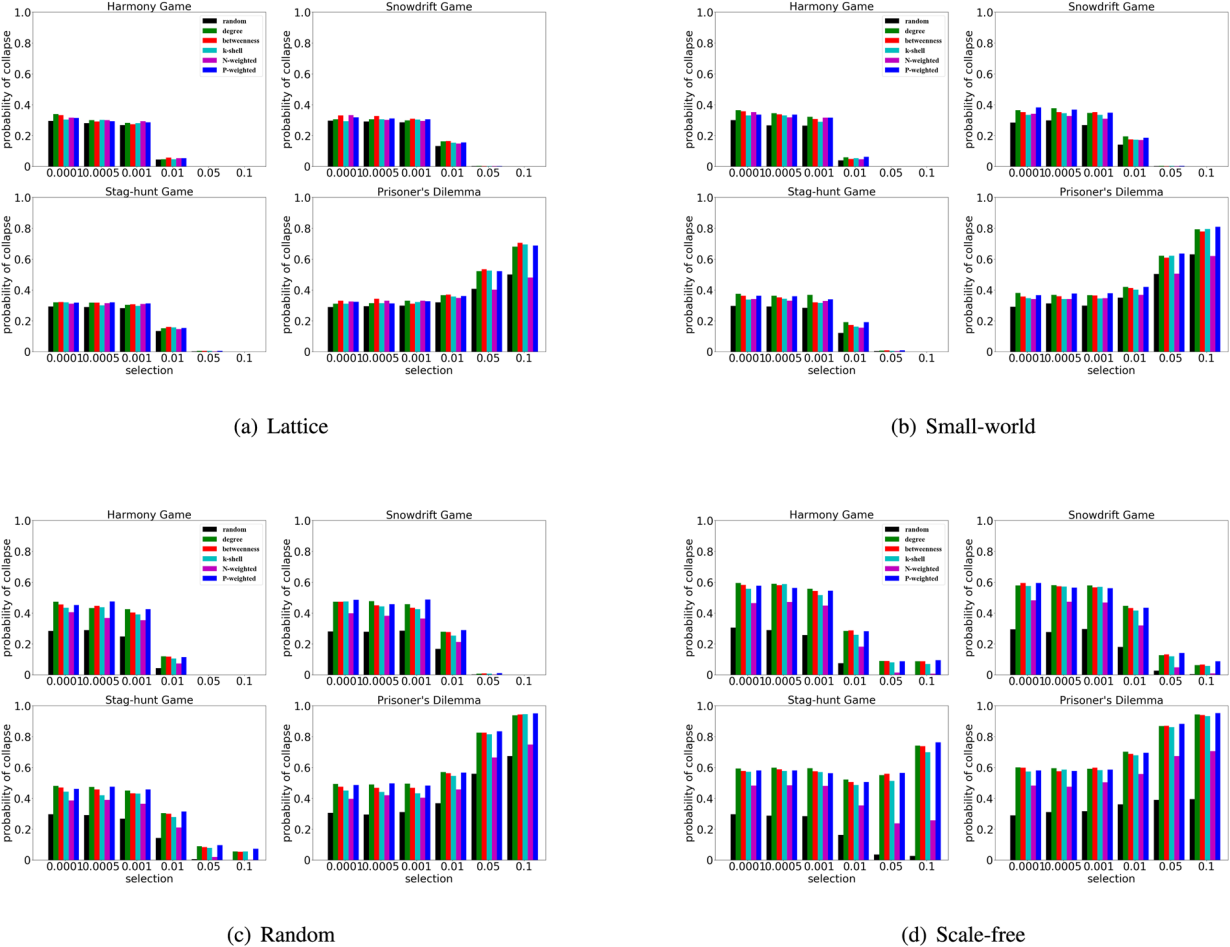
The collapse of cooperation depends on the selection strength, rankings and network structures. We use an initial density of invading cheaters $\rho =30 \%$, and the probability of collapse is obtained in a lattice, small-world, random and scale-free networks. For each subfigure, the x-axis indicates the strength of selection *w*, and the y-axis indicates the probability of cooperation collapse. We consider the effects of the different rankings - random, degree, betweenness, k-shell, N-weighted and P-weighted ranking - for four typical games.

In Fig. 5 we focus on the effects of selection on the efficacy of the rankings. In fact, we can observe that for very weak selection ($w=0.0001,0.0005,0.001$) the difference between the efficacy of the rankings is small, especially in lattice and small-world networks. On the other hand, more structured rankings facilitate the collapse of cooperation in random and scale-free networks (Fig. 5). We can also observe that in the harmony game ($S\in [0,1]$ and $T\in [0,1]$), snowdrift game ($S\in [0,1]$ and $T\in [1,2]$) and stag-hunt game ($S\in -\,1,1]$ and $T\in [0,1]$), the chances of cooperation collapse decrease as the selection strength gets stronger, while in the prisoner’s dilemma ($S\in [\,-\,1,0]$ and $T\in [1,2]$) the chances of cooperation collapse increase with the increase of the selection strength. In particular, for the stag-hunt game in scale-free networks (Fig. 5(d)), we can observe two opposite interesting trends: (i) the chances of cooperation collapse decrease with the increase of selection strength for the rankings based on random and N-weighted degree; (ii) the chances of cooperation collapse increase with the increase of selection strength for the rankings based on degree, P-weighted degree, betweenness.

**Figure 6:**
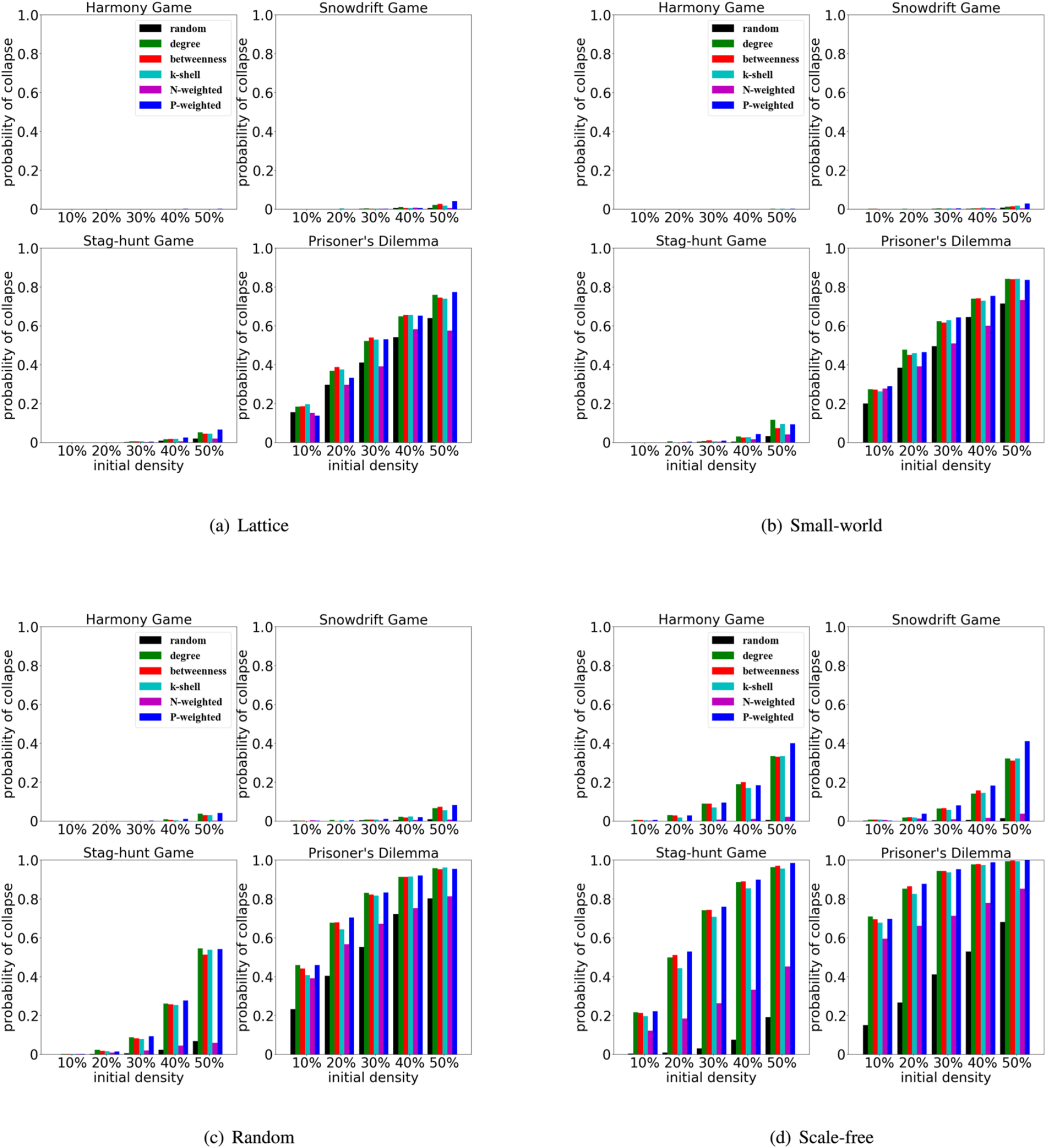
The collapse of cooperation depends on the initial densities, rankings and network structures. We use a medium selection at $w=0.05$, and the probability of collapse is obtained in a lattice, small-world, random and scale-free networks. For each subfigure, the x-axis indicates the initial density $\rho$, and the y-axis indicates the probability of cooperation collapse. We consider the effects of the different rankings - random, degree, betweenness, k-shell, N-weighted and P-weighted ranking - for four typical games.

Furthermore, we also explore the influence of initial density on the collapse of cooperation. With the increase of the initial fraction of cheaters, namely $\rho$, we find that the ranking based on P-weighted degree performs increasingly better (Fig. 6), especially for the harmony games and snowdrift games in scale-free networks.

## The Effects of Rankings in Scale-Free Networks

**Figure 7:**
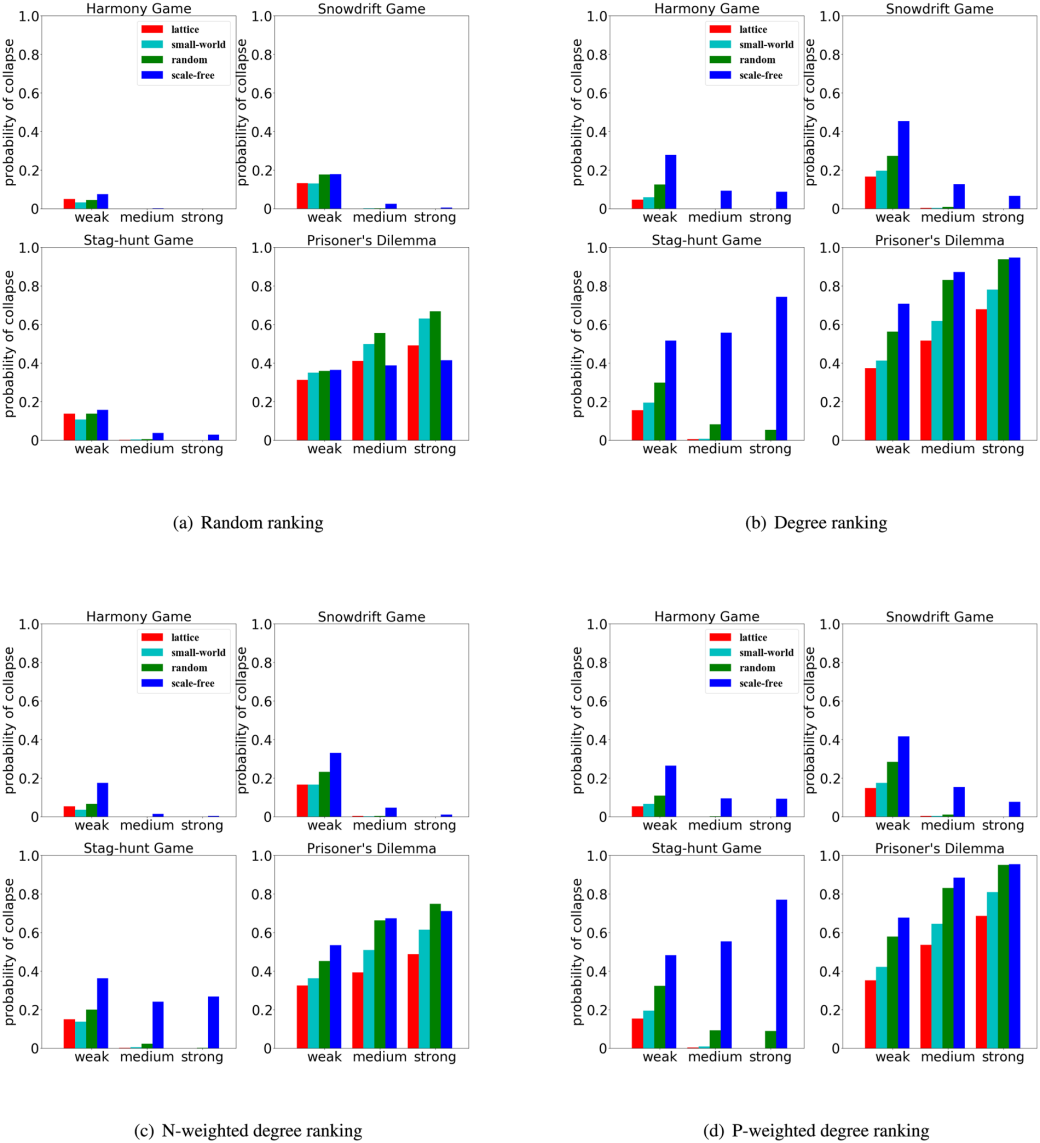
The effects of population structures on cooperation collapse depend on the game, selection strength and rankings. We use an initial density of cheaters $\rho =30 \%$ and compute the probability of cooperation collapse in a lattice, small-world, random and scale-free networks at weak selection ($w=0.01$), medium selection ($w=0.05$) and strong selection ($w=0.1$). We consider the effects of four ranking strategies - random ranking, the degree ranking, the N-weighted degree ranking and the P-weighted degree ranking.

As we can see in Fig. 7 the chances of cooperation collapse are usually maximized when the population is organized as scale-free networks, when an appropriate ranking (which facilitates the selection of hubs as initial cheaters) is used. However, as we can see in Fig. 7, the appropriate ranking which maximizes the chances of cooperation collapse in scale-free networks is not unique. In fact, for the prisoner’s dilemma (with $S=-\,0.4$ and $T=1.4$), random ranking or N-weighted degree ranking applied to scale-free networks perform worse (i.e., lower chances of cooperation collapse) than the same rankings in the other types of networks such as small-world networks (Fig. 7(a,c)); however the chances of cooperation collapse are maximized in scale-free networks when degree ranking or P-weighted degree ranking is used (Fig. 7(b,d)).

## The Effects of Selection on Rankings

**Figure 8:**
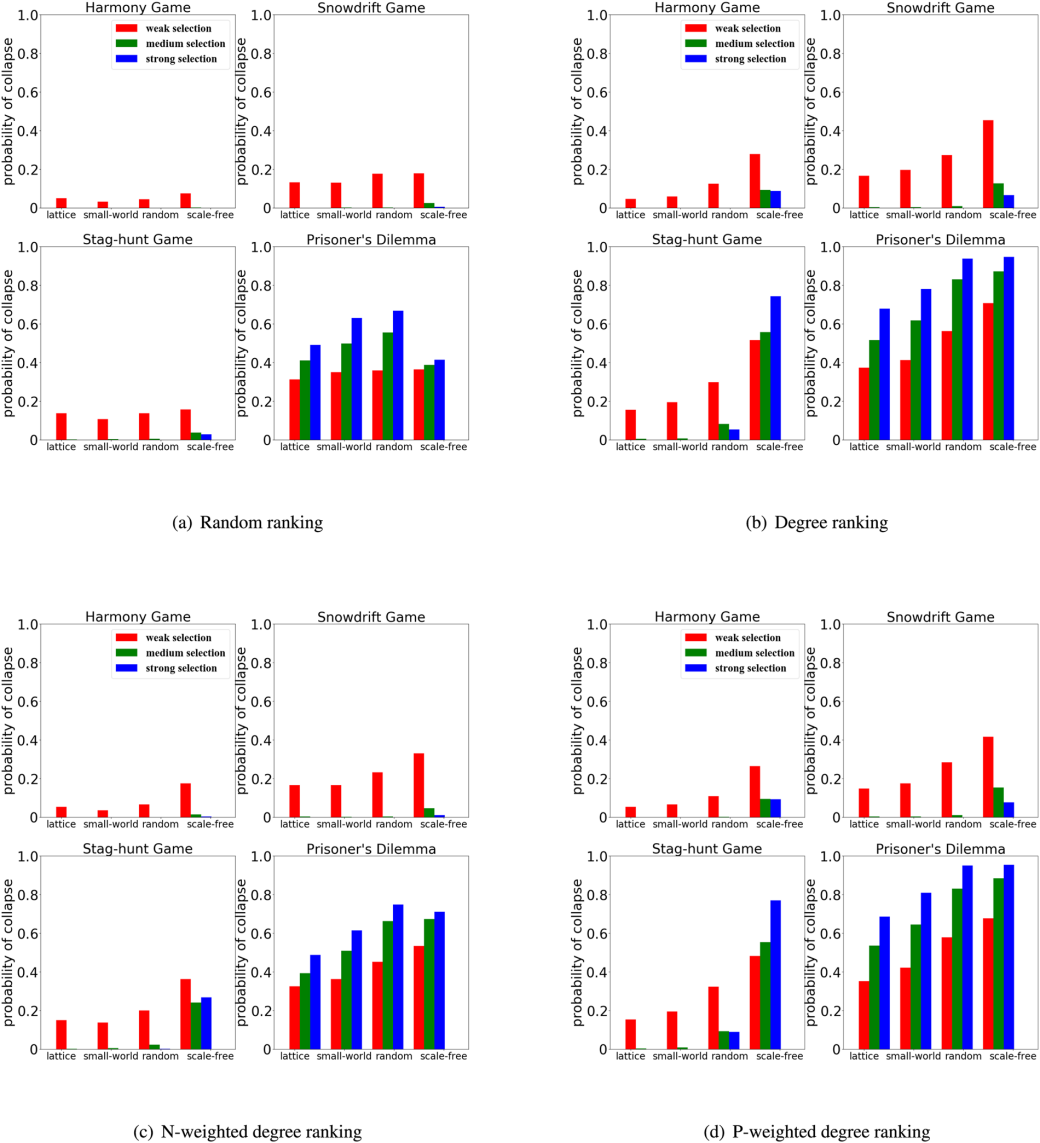
The effects of selection on cooperation collapse depends on the game, network structure and ranking. We fix an initial density of invading cheaters as $\rho =30 \%$ and compute the probability of collapse in a lattice, small-world, random and scale-free networks at weak selection ($w=0.01$), medium selection ($w=0.05$) and strong selection ($w=0.1$). We compare four ranking strategies - random ranking, the degree ranking, the N-weighted degree ranking and the P-weighted degree ranking.

Because of the way we compute the fitness, a stronger selection strength will amplify payoffs differences, while a weaker selection will reduce such difference. As we can see in Fig. 8 the selection strength has also an important effect on the performance of the rankings. For some regimes of the harmony game ($S\in [0,1]$ and $T\in [0,1]$), snowdrift game ($S\in [0,1]$ and $T\in [1,2]$) and stag-hunt game ($S\in [\,-\,1,0]$ and $T\in [1,2]$) the chances of cooperation collapse are weakened at strong selection, which is essentially caused by the relative big payoff obtained by the the cooperators. On the other hand, for large part of the prisoner’s dilemma regime ($S\in [\,-\,1,0]$ and $T\in [1,2]$), the payoff of defectors will be big, leading to higher chances of cooperation collapse when selection is strong.

Furthermore, we can also observe that the chances of cooperation collapse decreases when it is employed a random ranking or the N-weighted degree ranking in the case of stronger selection, but increases when an appropriate structured ranking is employed (e.g. the degree ranking or the N-weighted degree ranking), especially in scale-free networks (Fig. 8).

The stag-hunt game (with $S=-\,0.4$ and $T=0.6$) represents well this dual role of selection on rankings performance. Increasing selection leads to a smaller probability of cooperation collapse when either the random ranking or the N-weighted degree ranking is employed (Fig. 8(a,c)), but, at the same time, increasing selection leads to higher chances of cooperation collapse when the degree and P-weighted degree rankings are used in scale-free networks (Fig. 8(b,d)).

## Random Ranking vs Structured Rankings

The random ranking is the typical one used in literature - in many cases the resilience of cooperation is studied by adding invaders in randomly chosen positions. However, as we have seen, the chances of the collapse of cooperation may be very different if more structured rankings are employed, i.e., invaders are not placed randomly but according to some strategic choice.

**Figure 9:**
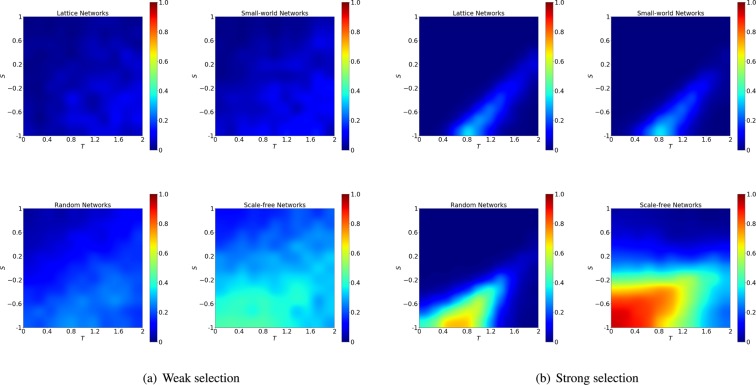
Performance of random ranking vs structured rankings. We plot the difference in the chances of cooperation collapse between the random ranking and the highest performance obtained by the best structural ranking. The biggest difference is obtained in the stag-hunt game and at strong selection. We use an initial density of invading cheaters $\rho =30 \%$ and plot the difference between the best structured ranking and the random ranking for weak selection with $w=0.01$ (left panels) and strong selection with $w=0.1$ (right panels). We consider population organized on a lattice, small-world, random and scale-free networks. For each subfigure, the x-axis indicates the game parameter $T\in [0,2]$ and the y-axis indicates the game parameter $S\in [\,-\,1,1]$.

As we can see in Fig. 9 the difference between the chances of cooperation collapse in the case of random ranking and in the case of more structural ranking crucially depend on the population structure, the game and the strength of selection. We explore in details how the difference of performance (i.e. cooperation collapse) between the best ranking and the random ranking depends on the network structures (lattice, small-world networks, random networks, scale-free networks) in the case of weak selection ($w=0.01$) and strong selection ($w=0.1$). As shown in Fig. 9 the difference in the performance is highly dependent on the structures of networks as well as the strength of selection.

At weak selection, the difference between the performance of the best ranking and the random ranking is only particularly large in the case of scale-free networks. However, at strong selection, the difference between the performance of the random rankings and the others is more evident - such difference reaches the maximum when the network is scale-free, in the regime corresponding to $S\in [0,1]$ and $T\in [\,-\,1,0]$ (stag-hunt game). The intuition behind such big gap between the performance of the random ranking and the others is that when selection strength is strong, and there is large degree heterogeneity in the network, the large payoffs of high-degree defectors will be enhanced in the competitions with cooperators by the strong selection, so that the rankings based on structured strategies (and which tend to select more connected nodes as initial cheaters) can outperform the random ones where initial cheaters are randomly chosen (independently of their position).

## Discussion

In dynamically evolving structured populations, the competition between cooperation and defection determines the stability of the system. Introducing some defectors in a cooperative communities may trigger the collapse of cooperation. However, it is not known how to identify influential invaders. e.g., those defectors which can mostly lead to the collapse of cooperation. In this paper, we analyze for the first time to our knowledge, the methodologies (rankings) which can be used to identify influential invaders when the population is organized on static complex networks. We combine evolutionary graph theory with ranking methodologies (used in epidemics) to demonstrate that the there is no optimal ranking but this depends on the population structure, the underlying game and the strength of selection. We compare some well-known rankings and show that the random ranking (i.e., where invaders are placed randomly) performs worse than structured rankings (where invaders are placed according to some structural strategy). The ranking based on degree can successfully identify the most influential invaders when selection strength is weak, while the ranking based on P-weighted degree performs better when selection strength is strong. The efficacy of the rankings is also affected by the typology of network which structures the population. Scale-free networks and strong selection may be detrimental to the performance of the random ranking, but will be beneficial to the performance of certain structured rankings (such as degree ranking and the P-weighted degree ranking). The random ranking generally performs worse than the other rankings, however the difference between its performance and structured rankings depend on the game, strength of selection and network structure. Such difference is the largest when the underlying game is the stag-hunt, population is organized in a scale-free network and selection strength is strong. These results demonstrate that when studying the collapse of cooperation in structured populations is important to study in details the mechanisms by which cheating invaders can appear in the population - not only their number, but also their initial positions can crucially affect the ability of cooperation to resist the invasion. Different ways to rank the individuals suggest different methodologies to distinguish the most influential invaders - some rankings perform better than others; there is however not a general optimal way to rank the invaders but the performance of the rankings depend on the careful study of the way the population is structured, the strength of the competition and the underlying game dynamics. We have applied the idea of rankings to a well-known evolutionary model such as evolutionary graph theory but the proposed methodology can be naturally extended to different models of structured populations^40^ leading to a novel promising research area.

## Methods

While degree centrality only considers the number of nearest neighbours, the *k-shell* of a node is defined in^11^ as a way to measure the location of a node as well as its degree. In k-shell decomposition, all isolated nodes have coreness ${c}_{0}=0$, and then all nodes with degree 1 are removed, causing a reduction of the degree values for the remaining nodes. The main idea is to keep removing nodes with degree of 1 until the degree of the remaining nodes is bigger than 1. The coreness of the removed nodes at round 1 is defined to be ${c}_{1}=1$, while the coreness of the nodes removed at round *t* is defined to be ${c}_{t}=t$. A larger coreness for a node means that the corresponding node is located in a more central position (i.e., it has a higher k-shell).

The weighted degree decomposition (WDD) is defined in^36^ as a way to compute the importance of nodes by considering the difference between the removed nodes and the remaining nodes. In general, two types of nodes will be in the network according to the weighted degree decomposition method, and they are defined to be seed nodes and potential nodes. These are defined in the following way:

- a seed node - a node selected into the *top-n-set* as one of the initial cheating invaders;
- a potential node - a node that is not in the *top-n-set*.

We compute the weighted degree of a node as follows:

3 $${k}^{\ast }(v)={k}_{p}(v)+{\Sigma }_{u\in NS(v)}(\alpha +\beta |NS(u)|)$$

where ${k}_{p}(v)$ is the number of potential nodes in the neighbourhood, and *NS*(*v*) is the set of neighbours who have been selected into the *top-n-set* as seed nodes. There are two weight coefficients *α* and *β* to allow a trade-off between exploitation and exploration. Depending on the values of *α* and *β*, we have the N-weighted degree ranking or the P-weighted degree ranking.

For the N-weighted degree ranking, the seed neighbours as well as their seed neighbours will be under-rated (i.e., associated to negative coefficients in the calculation of the weighted degree decomposition). In order to avoid the overlap of sphere of influence, *α* and *β* will be set to be negative, and the neighbours’ degree of the seed nodes will be discounted; this will also lead to the selection of nodes which are far away from the crowd of selected nodes.

For the P-weighted degree ranking, however, its seed neighbours as well as their seed neighbours will be over-rated ((i.e., associated to positive coefficients in the calculation of the weighted degree decomposition). Both *α* and *β* will set to be positive; this will lead to the selection of nodes close to the cluster of high-degree seeds.

The selection of the n initial invaders by using weighted degree decomposition is generally implemented with the following procedure: (1) the node with the maximum weighted degree is selected and added into the *top-n-set*; (2) the states of nodes in the neighbourhood of the selected node are updated and the weighted degree for the remaining nodes is recomputed; (3) repeat the above steps until the size of *top-n-set* reaches *n*.
